# Prevalence and determinants of common mental health illnesses among reproductive-aged women in Bangladesh: Evidence from Demographic and Health Surveys data 2022

**DOI:** 10.1371/journal.pmen.0000352

**Published:** 2026-02-19

**Authors:** Afsana Mimi

**Affiliations:** Department of Statistics, University of Dhaka, Dhaka, Bangladesh; University of Connecticut, UNITED STATES OF AMERICA

## Abstract

Women are more vulnerable to depression and/or anxiety comprised as common mental illnesses (CMIs) worldwide, particularly in Bangladesh. To date, no study has conducted in broad scale to explore the associated factors with CMIs among women in Bangladesh. This study aims to identify the significant strength of association of several factors with the presence of CMIs, which are responsible for the development of mental stress. To accomplish this work, Bangladesh Demographic and Health Survey (BDHS) 2022 data have been used, are publicly available in the Demographic and Health Surveys (DHS) program. A total of 19985 ever-married women aged 15–49 years are incorporated in this work. The association of selected predictors with mental health illness among women was assessed using Chi-square test and a binary logistic regression model was applied to explore the effect of those predictors on the presence of mental health illnesses. The study explored that 20.4% of reproductive-aged women have suffered from common mental illnesses (CMIs). Women aged 25–35 years and above 35 years have higher odds of experiencing CMIs [Odds = 1.46, Cl: 1.30-1.63; Odds = 1.78, CI: 1.58-2.00]. The women who belong to Barishal, Chattogram, Khulna, Rangpur, and Sylhet have 1.17 [CI: 1.00-1.36], 1.27 [CI: 1.11-1.46], 1.35 [CI: 1.17-1.56], 1.62 [CI: 1.41-1.87], and 1.15 [CI: 0.99-1.34] times higher odds of having CMIs. Women who have lost their pregnancies have 28.3% higher odds of having the CMIs [CI: 1.18-1.39]. The estimated odds for women who suffered from both social obstacles and social violence are 1.58 [CI: 1.45-1.71] and 1.27 [CI: 1.15-1.41], respectively. These results suggest that women should receive focused mental health interventions, especially those who are dealing with social violence, pregnancy losses, and other psychosocial issues. Improving women’s access to economic and educational possibilities can also act as a buffer against common mental illnesses.

## Introduction

Mental health has emerged as the emotional, cognitive, and physical well-being, specifically among reproductive-aged women in low and middle-income countries [1,2]. Approximately one in eight persons worldwide suffers from a mental illness, with depression and anxiety disorders being the most common mental health conditions [3]. It is estimated that 5.0% of adults worldwide experience depression, affecting around 280 million individuals of all ages [4,5]. Women are predominantly diagnosed with depressive disorders, with an estimated 201.3 million women affected globally, in contrast to 131.1 million males [6]. Anxiety disorders are one of the most common mental health issues, affecting 301 million individuals worldwide, with 4.6% in women and 2.6% in men, highlighting the disproportionate burden of anxiety among women [7,8]. These common mental diseases (CMIs) have affected more than 14% of women between the ages of 15 and 49 [9].

People suffering from CMIs often experience higher incidences of other diseases. According to Gross et al, the risk of chronic diseases such as obesity, hypertension, diabetes, and cardiovascular disease increases with the presence of common mental illnesses (CMIs), with similar findings reported by Pan et al [10,11]. Other studies have found that the immune system function could be impaired by CMIs, increasing susceptibility to infections [12,13]. Katon explored that people with CMIs frequently suffer from gastrointestinal problems, sleep disorders, chronic headaches, and exhaustion, despite the absence of a biological cause [14]. Over 700,000 deaths globally are attributed to depression, making it one of the primary causes of suicide [15]. The risk of suicide is also greatly increased by anxiety disorders, particularly when they coexist with depression [16,17]. As a coping strategy, people with CMIs are more likely to abuse illegal drugs, alcohol, or tobacco [18]. Depression can affect focus, decision-making, and memory, and these issues may persist even after mood symptoms subside [19]. According to Whisman, social disengagement, poor communication, and relationship problems, such as marital friction and family disintegration, are frequently caused by CMIs [20]. Children’s behavioral issues, developmental delays, and insecure attachment are all linked to maternal CMIs [21–23]. Preterm birth, low birth weight, poor infant health, and an increased risk of postpartum illnesses that can hinder caregiving and maternal-infant attachment could all be consequences of maternal CMIs [24,25].

Numerous research had been carried out in different nations to investigate the contributing factors in order to better understand and manage the effects of common mental illnesses. For instance, Hossain et al. reported that 30% of rural women have suffered from mental distress [26]. Other researchers have identified increasing age as a significant factor contributing to common mental illnesses (CMIs), suggesting that the likelihood of developing CMIs rises with age [27,28]. Additionally, women’s wealth status and educational attainment have been significantly associated with the presence of anxiety and depression, as reported in several studies [27,29–32]. Regional disparities in women’s mental health have also been documented, often attributed to uneven economic development, limited recreational opportunities, and restricted access to healthcare services, as reported by several researchers [33,34]. Moreover, studies have found that financially independent women are less likely to experience CMIs [35]. Migrant women, however, are more vulnerable to anxiety and depression due to the challenges of adapting to new environments [36]. Further research highlights that women who have experienced pregnancy loss or intimate partner violence face a significantly higher risk of mental health issues [37–45]. Barriers to accessing healthcare—such as distance, cost, or social constraints—also play a major role in the development of CMIs [46]. Acharya et al. explored that greater autonomy is associated with lower levels of metal distress [47].

Despite the growing evidence base, significant gaps remain in understanding the contextual determinants of mental health among reproductive women, specifically in Bangladesh. The BDHS 2022, with its inclusion of mental health indicators, presents a unique opportunity to address some of these gaps. This study will explore how structural inequalities such as region, or policy implementation, interact with psychosocial variables to influence mental disorders.

## Data source

**Fig 1:**
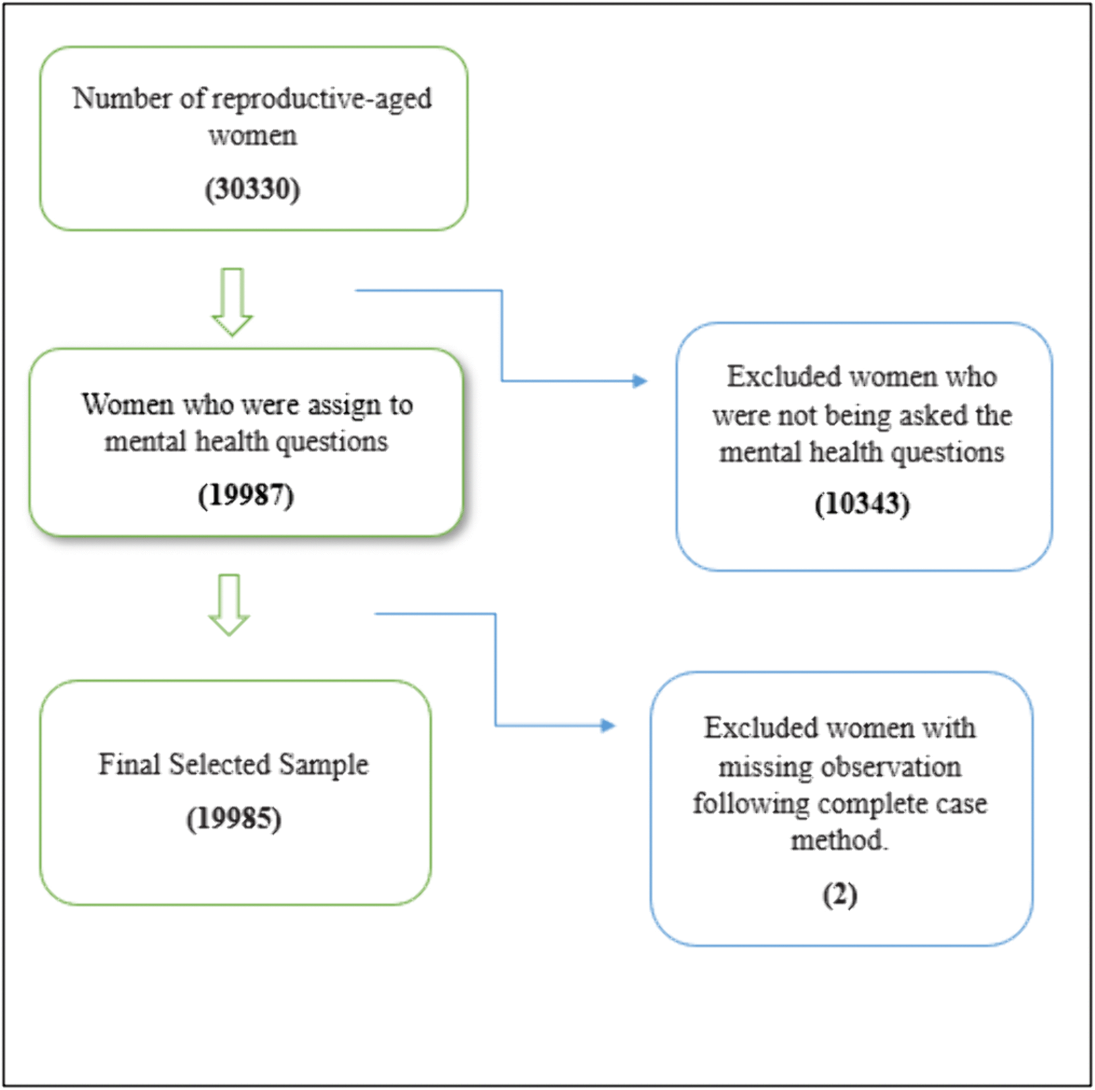
The final sample size selection.

This study utilized nationally representative secondary data, the Bangladesh Demographic Health Survey Data 2022, conducted by National Institute of Population Research and Training (NIPORT), Mitra and Associates, & ICF. [48]. It is the ninth cross-sectional DHS survey comprising a two-stage cluster sampling design, where in the initial stage, a total of 675 enumeration areas (EAs) were selected (438 from rural regions and 237 from urban ones), using probability proportional to the size of the population. The detailed sampling procedure and methodology were well-described in BDHS 2022 report [49]. The eligible respondents of this survey were ever-married women aged between 15 and 49 years old. The survey questionnaire was designed in a long and short version of the individual questionnaire. The long version questionnaire, which included mental health-related questions for women, was administered to a random sample of 30 households among 45 households in each EA. Finally, to avoid the adverse situations, the survey was carried out with 674 clusters, excluding one rural EA, resulting in a total sample of 30330 households, and 19987 women were allocated to mental health questions that have been utilized to serve the purpose of this study. Finally, a total of 19985 unweighted observations were used to perform statistical analysis after excluding cases with missing observations following the complete case analysis approach. A flowchart is represented to describe the inclusion and exclusion of the observation in Fig 1.

### Outcome variable

This study defines common mental illnesses (CMIs) as a binary outcome that captures whether a woman has symptoms of depression or anxiety. If women met the criteria for either disease, they were labeled as having CMIs.

The Generalized Anxiety Disorder-7 (GAD-7) scale, which consists of seven questions that represent the anxiety symptoms experienced during the preceding two weeks, was used to measure anxiety symptoms. With a total score range of 0–21, each item is scored on a 4-point scale ranging from 0 (“not at all”) to 3 (“nearly every day”). According to Spitzer et al., anxiety symptoms are present when the score is six or higher [50].

The Patient Health Questionnaire-9 (PHQ-9), which consists of nine items assessing depressive symptoms during the previous two weeks, was used to quantify depressive symptoms. Additionally, each item is given a rating from 0 (“not at all”) to 3 (“nearly every day”), for a total score between 0 and 27. According to Kroenke et al., respondents with a score of 10 or higher were deemed to be depressed [51].

### Covariates

A wide range of demographic and socioeconomic factors, pregnancy history and psychosocial factors of women is included in the analysis based on literature [27–47]. Three age groups of reproductive women are distinguished: those under 25, those between 25 and 35, and those over 35. Primary education, secondary education, higher education, and no formal education (illiteracy) are the four categories of women’s educational attainment that have been considered. The eight administrative divisions of Bangladesh—Dhaka, Barishal, Chattogram, Khulna, Mymensingh, Rajshahi, Rangpur, and Sylhet—determine the geographic location, whereas the place of living is classified as either urban or rural. Household wealth indices are used to divide economic status into three categories: poor, middle, and rich. Women are categorized as either currently unemployed, having worked within the last 12 months, or currently employed to assess their employment situation. Furthermore, a variable that indicates if a woman has lost any pregnancies throughout her reproductive years is used to record her reproductive history. The ability to freely or collaboratively participate in decision-making concerning any of the following areas is referred to as women’s autonomy: (1) visiting healthcare services, (2) spending money, (3) purchasing household, and (4) visiting family or relatives. If a woman participates in decision-making in at least one of these areas, she is deemed to have autonomy. The three key covariates: (1) whether permission is needed to go outside (No problem/Big problem), (2) whether money is needed for treatment (No problem/Big problem), and (3) the distance to medical facilities (No problem/Big problem)—are combined to create the binary indicator known as the social obstacle variable. The existence of any logistical or social obstacles that can prevent people from accessing healthcare services is captured by this variable. Four observable indicators are utilized to form a binary variable that represents domestic violence: (1) being physically hurt for burning food, (2) being abused for neglecting children, (3) experiencing violence following a disagreement with a husband, and (4) being abused for refusing to engage in sexual relations. A person is classified as having experienced domestic abuse if they answer “Yes” to any of these scenarios. These two covariates, social obstacles and domestic violence, are attributed as psychosocial variables.

### Ethics approval

Demographic and Health Surveys (DHS) Program, including the 2022 Bangladesh DHS, was examined and authorized by the Institutional Review Board of ICF International, Rockville, Maryland, USA (previously Macro International). The Bangladesh Medical Research Council also approved the Bangladesh DHS for 2022. Under the direction of the Government of the People’s Republic of Bangladesh, the National Institute of Population Research and Training (NIPORT) carried out this survey with funding assistance from USAID/Bangladesh. Every survey respondent gave their informed consent before participating, and those who declined were excluded from the study. No personal identifiers were included in the data before it was made publicly available. The data are publicly available on the DHS program website (https://dhsprogram.com/Data/terms-of-use.cfm).

### Statistical analysis

This study aims to identify the significant covariates, which are responsible for developing CMIs among the eligible women aged 15–49 years old in Bangladesh, as well as significant factors that play an important role in reducing mental distress. Following this, thoroughly univariate, bivariate, and multivariable analyses are conducted. In univariate analysis, the frequency with percentage of each covariate along with the outcome variable is observed. Further, a chi-square test is applied to find out whether the association between selected covariates and the presence of CMIs exists in bivariate analysis [52]. Finally, the significance and strength of association of selected factors with CMIs among women are assessed by applying a binary logistic regression model [53]. The overall analyses are carried out using IBM SPSS Statistics version 27 which provides reliable methods for both descriptive and inferential statistics and is often utilized and validated for comparable kinds of research.

## Results

### Univariate analysis

The percentage and count of all covariates, including socio-economic, pregnancy history, psychosocial, and outcome variable, defined as mental illness status, are presented in Table 1. The percentage of women who experienced mental stress is 20.4%, indicating the prevalence of anxiety and depression among women observed in Bangladesh. Among all ages, 41.1% of women are above 35 years old, and most women are secondarily educated (45.3%). The percentage of wealthier women is high (42.3), as well as for those who are unemployed (64.1).

**Table 1:** Distribution of sample observation.

Variables	Percentage	Count
**Mental illness status**		
No	79.6	15903
Yes	20.4	4082
**Respondent’s age in years**		
Below 25	24.3	7300
25-35	34.7	10427
Above 35	41.1	12351
**Level of education**		
No education	13.9	4167
Primary education	26.1	7857
Secondary education	45.3	13613
Higher education	14.8	4441
**Place of residence**		
Rural	64.9	19507
Urban	35.1	10571
**Division**		
Dhaka	15.1	4554
Barishal	10.7	3232
Chattogram	14.8	4461
Khulna	13.1	3928
Mymensingh	10.8	3255
Rajshahi	12.7	3816
Rangpur	12	3624
Sylhet	10.7	3208
**Wealth index**		
Poor	37.9	11414
Middle	19.7	5926
Rich	42.3	12738
**Working status**		
No work	64.1	12802
Work in the last year	5.1	1010
Working	30.9	6175
**Living with husband**		
No	53.5	16101
Yes	46.5	13977
**Migration**		
No	79	23769
Yes	21	6309
**Pregnancy losses**		
No	76.6	23341
Yes	22.4	6737
**Autonomy**		
No	12.5	2364
Yes	87.5	16623
**Social obstacle**		
No	56.2	16901
Yes	43.8	13177
**Domestic violence**		
No	87	17372
Yes	13	2607

### Bivariate analysis

To identify whether there is a significant relationship between mental illnesses and psychosocial, as well as socio-demographic factors, and pregnancy outcome variables, chi-square analysis was utilized, and the results of the associations with p-values are displayed in Table 2.

**Table 2:** Percentage of common mental illnesses (CMIs) among Bangladeshi reproductive-age women between 15 and 49 years old.

Covariates	Mental Illness		Chi-Square	*p*-value
	Yes (%)	No (%)		
Respondent’s age in years			231.71	<0.001
Below 25	680 (14)	4187 (86)		
25-35	1368 (19.6)	5609 (80.4)		
Above 35	2034 (25)	6107 (75)		
Level of education			184.80	<0.001
No education	752 (27.6)	1969 (72.4)		
Primary education	1190 (22.9)	4015 (77.1)		
Secondary education	1717 (18.8)	7419 (81.2)		
Higher education	423 (14.5)	2500 (85.5)		
Place of residence			2.987	0.084
Rural	2698 (20.8)	10281 (79.2)		
Urban	1384 (19.8)	5622 (80.2)		
Division			76.12	<0.001
Dhaka	529 (17.5)	2499 (82.5)		
Barishal	420 (19.8)	1697 (80.2)		
Chattogram	657 (22)	2326 (78)		
Khulna	567 (21.8)	2034 (78.2)		
Mymensingh	377 (17.5)	1779 (82.5)		
Rajshahi	475 (18.7)	2070(81.3)		
Rangpur	607 (25.3)	1792(74.7)		
Sylhet	450 (20.9)	1706(79.1)		
Wealth index			47.08	<0.001
Poor	1713 (22.8)	5787 (77.2)		
Middle	799 (20)	3190 (80.0)		
Higher	1570 (18.5)	6926 (81.5)		
Working status			14.65	<0.001
No work	2510 (19.6)	10290 (80.4)		
Work in the last year	218 (21.6)	792 (78.4)		
Working	1354 (21.9)	4821 (78.1)		
Living with husband			18.30	<0.001
No	3192 (19.8)	12908 (80.2)		
Yes	890 (22.9)	2995 (77.1)		
Migration			54.1	<0.001
No	3395 (21.5)	12387 (78.5)		
Yes	687 (16.3)	3516 (83.7)		
Pregnancy losses			61.33	<0.001
No	2941 (19.2)	12382 (80.8)		
Yes	1141 (24.5)	3521 (75.5)		
Autonomy			11.81	<0.001
No	399 (16.9)	1965 (83.1)		
Yes	3303 (19.9)	13319 (80.1)		
Social obstacle			157.46	<0.001
No	1052 (15.4)	5758 (84.6)		
Yes	3030 (23)	10145 (77)		
Domestic violence			47.08	<0.001
No	3415 (19.7)	13955 (80.3)		
Yes	664 (25.5)	1943 (74.5)		

From Table 2, the association between the age of women and the presence of CMIs appears significant at a 1% level of significance (p < 0.001), highlighting that the risk of having CMIs increases with age, notably older women have the highest percentage (25%) of CMIs. On the other hand, the educational attainment of women has a great influence on reducing the risk of CMIs among women; highly educated women have the lowest percentage (14.5%) of experiencing mental stress. Common mental health disorders among women from Rangpur have the highest percentage (25.3%), while among the women from both Dhaka and Mymensingh appear to have the lowest percentage (17.5%). On the other hand, wealthier women tend to have a lower risk (18.5%) of having CMIs. Interestingly, the highest percentages of CMIs are observed among women who live with their husbands (22.9%) and among women who are currently working or worked in the past year (21.9% and 21.6%, respectively). Moreover, migrant women tend to have a lower percentage (16.3%). In contrast, the highest percentages of CMIs were found among the women who had experienced pregnancy losses (24.5%), those who had faced social obstacles (23%), and those who had suffered from domestic violence (25.5%). Surprisingly, autonomous women have a higher risk of experiencing mental stress (19.9). In short, all covariates are significantly associated with the presence of CMIs except the place of residence of women.

### Binary logistic regression model

The significance and the strength of association of socio-demographic factors, autonomy, social obstacles, social violence, and other variables on the presence of mental illnessess among reproductive-age women (15–49 years) in Bangladesh are assessed by implementing a binary logistic regression model, as the outcome variable is defined as a binary outcome. To achieve this goal, the model’s results, odds ratio (OR), 95% confidence interval, and *p*-value were analyzed and presented in Table 3. Additionally, the model goodness of fit test is evaluated by likelihood ratio chi-square test and the result presented in Table 3. The model is well-fitted as the test is highly significant at 1% level of significance.

**Table 3:** Determinants of mental disorders among reproductive-age women (15-49 years) using a binary logistic regression model.

Covariates	Odds Ratio (OR)	95% Confidence Interval (CI)	p-value
Respondent’s age in years			
Below 25 (Ref)			
25-35	1.46***	[1.30, 1.63]	<0.001
Above 35	1.78***	[1.58, 2.00]	<0.001
Level of education			
No education (Ref)			
Primary education	0.86**	[0.77, 0.97]	0.012
Secondary education	0.78***	[0.69, 0.87]	<0.001
Higher education	0.62***	[0.53, 0.73]	<0.001
Place of residence			
Rural (Ref)			
Urban	1.04	[0.95, 1.12]	0.399
Division			
Dhaka (Ref)			
Barishal	1.17**	[1.00, 1.36]	0.044
Chattogram	1.27***	[1.11, 1.46]	<0.001
Khulna	1.35***	[1.17, 1.56]	<0.001
Mymensingh	0.94	[0.81, 1.10]	0.470
Rajshahi	1.04	[0.90, 1.20]	0.635
Rangpur	1.62***	[1.41, 1.87]	<0.001
Sylhet	1.15*	[0.99, 1.34]	0.070
Wealth index			
Poor (Ref)			
Middle	0.93	[0.84, 1.03]	0.152
Higher	0.88	[0.80, 0.97]	0.011
Working status			
No work (Ref)			
Work in the last year	1.05	[0.89, 1.24]	0.562
Working	0.96	[0.89, 1.05]	0.362
Living with husband			
No (Ref)			
Yes	1.01	[0.91, 1.12]	0.888
Migration			
No (Ref)		[0.91, 1.13]	0.839
Yes	1.01		
Pregnancy losses			
No (Ref)		[1.18, 1.39]	<0.001
Yes	1.28***		
Autonomy			
No (Ref)			
Yes	1.18*	[1.15-1.27]	0.070
Social obstacle			
No (Ref)			
Yes	1.58***	[1.45, 1.71]	<0.001
Domestic Violence			
No (Ref)			
Yes	1.27***	[1.15,1.41]	<0.001
Likelihood Ratio Chi-square Value	569.77***		**<0.001**

Ref means reference category, ***Significance at 1% level, ** Significance at 5% level, *Significance at 10% level

Women who were between 25 and 35 years old, and above 35 years old, both have higher odds of having CMIs compared to those who were below 25 years old. This is reflected in the odds ratio of 1.46 [CI:1.30-1.63] and 1.78 [CI:1.58-2.00], indicating that 45.5% and 77.7% higher odds of having CMIs for those aged 25–35 years and above 35 years, respectively, compared to those aged below 25 years, and these differences are statistically significant with a p value less than 0.001 for both. This result recommends that the vulnerability of havingCMIs increases with the age of women. Moreover, women’s educational attainment also has a significant association with the presence of mental disorders. In other words, the more women were educated, the fewer women had mental disorders. In particular, primary, secondary, and higher educated women have 0.86 [CI: 0.77-0.97], 0.78 [CI: 0.69-0.87], and 0.62 [CI: 0.53-0.73] times lower odds of having CMIs compared to illiterate women, and also indicating that a significant association among them with p-values are 0.012, < 0.001, and <0.001, respectively. Further, significant geographical differences have also appeared in the presence of CMIs among reproductive women aged 15–49. Particularly, women who belong to Barishal, Chattogram, Khulna, Rangpur, and Sylhet have 17%, 27.3%, 35%, 62%, and 15% higher odds of being vulnerable to CMIs, respectively, compared to those who belong to Dhaka, along with the p-value of below 0.001 for all. Notably, Rangpur’s women have the highest odds ratio of 1.62 [CI: 1.41-1.87], indicating that more prone to have CMIs. Furthermore, there is no significant association between CMIs and whether women belong to urban or rural areas. Similarly, the economic status and working status of women have no significant role in having CMIs. Mothers who have experienced pregnancy loss have 28.3% higher odds of having CMIs compared to those who have not experienced yet, highlighting a significant association with a p-value of 0.001 [Odds = 1.28, CI: 1.18-1.39]. Additionally, women who have faced social obstacles particularly in reaching out health care complex, and women who have suffered from social violence have 57.7% and 27.3% higher odds of having CMIs compared to those who have not experienced social obstacle and social violence both. These results reflect in the odds of 1.58 [CL1.45-1.71] and 1.27 [CI:1.15-1.41] respectively and both are significantly associated with a p-value of 0.001. Moreover, autonomy women have slightly association with the presence of CMIs (P-value = 0.070), indicating that women who have autonomy have 1.178 times higher odds of experiencing CMIs compared to those who have not. Migrant women as well as whether women were staying with husband have no significant association.

## Discussion

Using nationally representative data from BDHS 2022, this study offers important insights into the factors that contribute to common mental illnesses (CMIs), such as depression or anxiety, among Bangladeshi women of reproductive age. This study found that the prevalence of common mental illnesses among reproductive-aged women is 20.4% in Bangladesh, aligning with the result of the BDHS 2022 report [49]. The prevalence of CMIs is very low for Nepal [54]. In contrast, the prevalence is very high in rural Ethiopia [27]. Furthermore, the study found that age, education, division, pregnancy losses, autonomy, social obstacle, and social violence were significant associated covariates for developing CMIs.

The study emphasizes women’s age plays a significant role in experiencing CMIs by finding that odds of having CMIs increases with the age of reproductive women, indicating a positive association between age and the presence of CMIs; these findings align with other studies [27,28]. This pattern demonstrates an increase in mental health susceptibility with age, which could be brought on by chronic health conditions, caregiving responsibilities, or life stress. Further, this study has revealed that women’s educational attainment exerts a negative association with the presence of CMIs. This implies that the positive implication of educational attainment is on reducing CMIs, aligning with findings of another research works [27,30,54,55]. This finding can be attributed to the fact that a higher level of education enhances autonomy, coping strategies, and access to health-related information, resulting in a lower risk of experiencing CMIs.

Significant regional disparities have been observed in this study, indicating that women from Barishal, Chattogram, Khulna, Rangpur, and Sylhet have higher risk of experiencing CMIs compared to those from Dhaka. Particularly, women in Rangpur face the highest risk of mental vulnerability. In other nations, regional variations have also been noticed [33,34]. These consequences may happen due to the lack of social support, lower socio-economic conditions, limited access to healthcare services and educational resources, and uneven economic development across different regions [54,56]. Further, even though wealth status, working status, living with husband, and migration have no significant association with the experience of CMIs, they could be the sources of either support or mental stress based on the context of nature, such as legality and financial stability of a situation, job quality, satisfaction in the relationship, and the environment of new places [29,31,42,57].

Pregnancy outcome is a significant factor for CMIs, suggesting that women who have experienced miscarriage or stillbirth have risk of suffering from CMIs. This result indicates that the experiencing of CMIs is significantly associated with pregnancy losses, aligning with the evidence from both low- and high-income nations [39–41]. The most potent factors of CMIs found in this study are social obstacles and social violence among psychosocial covariates. These findings are consistent with worldwide evidence, which alarmingly highlights that social challenges and interpersonal violence significantly increase the risk of anxiety and depression in women aged 15–49 years [37,38,46]. These results highlight the importance of addressing sex-based discrimination and violence as an integral part of mental health policies.

Remarkably, there was only a weak association between autonomy and CMIs. This research suggests that autonomy on its own, without adequate emotional or structural support, may provide only a limited level of mental health protection [47]. In some cases, it could even make women more vulnerable, especially if having more autonomy entails having a greater responsibility or meeting social expectations.

## Conclusion

This research work underscores potential risk factors for experiencing common mental illnesses among reproductive-aged women in Bangladesh. The findings emphasize how critical it is to implement integrated mental health policies that go beyond clinical interventions to overcome social barriers, improve education and economic empowerment, address structural inequalities, and guarantee better healthcare services to lower pregnancy losses and gender-based violence. For significant improvements in mental health, policymakers should give priority to focused interventions in high-risk areas, among socioeconomically disadvantaged groups, and traumatized populations.

### Strengths and limitation

The study used nationally representative, high-quality, and publicly accessible BDHS 2022 data, which covers a variety of subjects. However, the data also has limitations: a large portion of the information is self-reported, which may add bias, and it is cross-sectional, making it impossible to prove causal links. Certain variables include incomplete or insufficient information on social and economic factors. In spite of these shortcomings, BDHS 2022 is still a reliable and powerful resource for health and demographic studies in Bangladesh.
